# Supplementary figures and images for: A unified physiological framework of transitions between seizures, sustained ictal activity and depolarization block at the single neuron level
Source: J Comput Neurosci. 2022 Jan 15;50(1):33–49. doi: 10.1007/s10827-022-00811-1 (PMC8818009; doi:10.1007/s10827-022-00811-1)

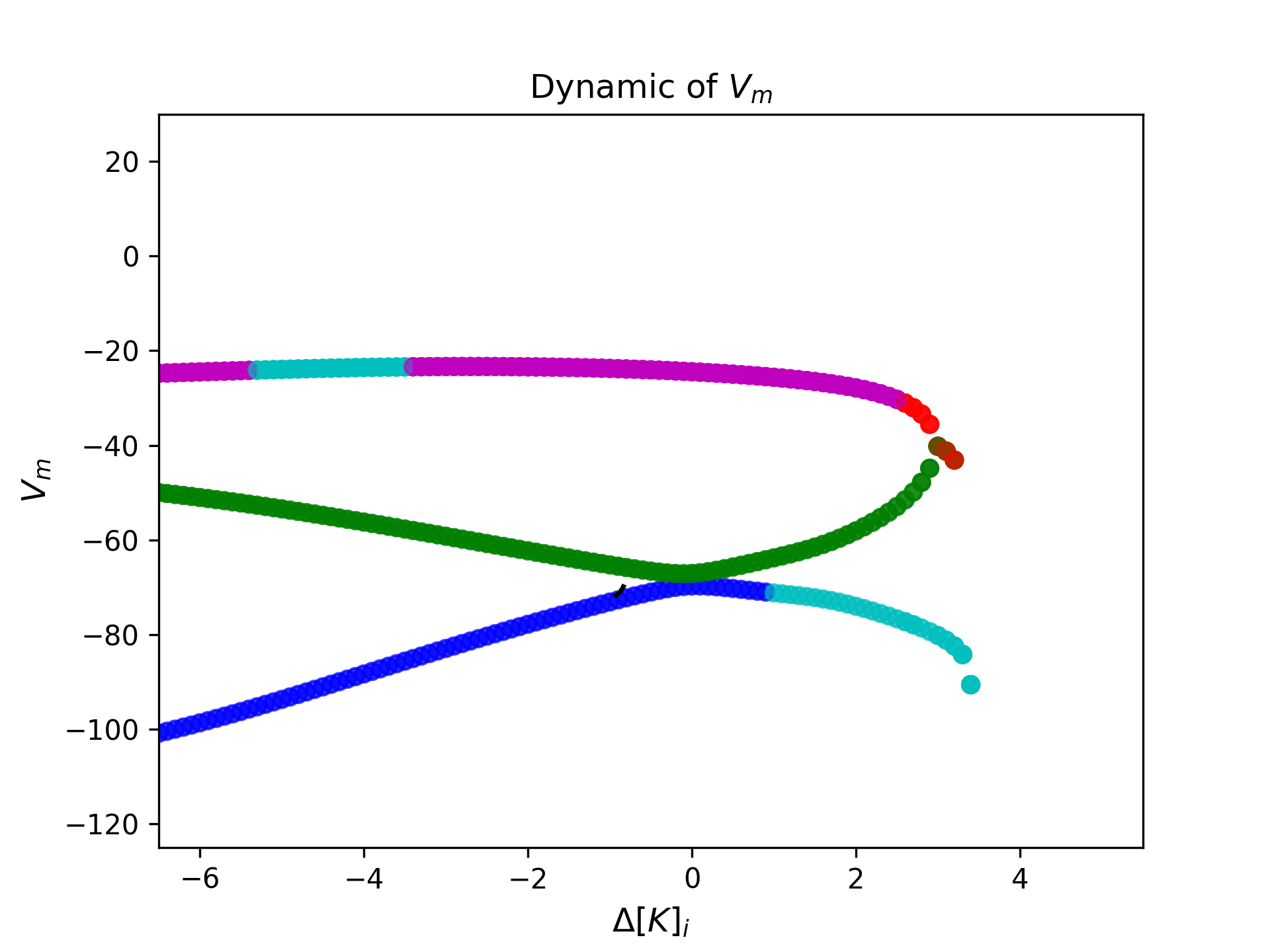

Supplement: Supplementary file 1 — Supplementary file1 S1 Animation. Dynamics of the membrane potential during burst. Considering the two slow variables as parameters of the fast subsystem, fixed point has been found: blue: stable node, green: saddle node, cyan: stable focus, magenta: unstable focus, red: unstable node. The system starts at a stable fixed point and is slowly driven to cross a saddle-node and then follow a limit cycle, until it cross again a saddle-node (creating the Homoclinic bifurcation), and go back to a stable fixed point. (GIF 1013 KB) [file 10827_2022_811_MOESM1_ESM.gif]

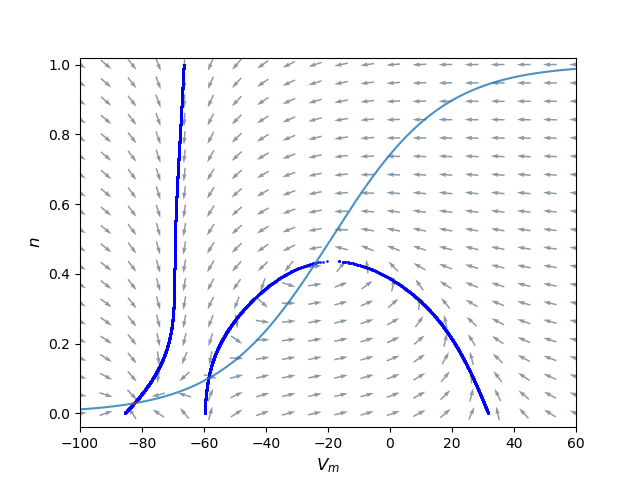

Supplement: Supplementary file 2 — Supplementary file2 S2 Animation. Dynamic during Burst observed in the phase plane. The n nullcline (blue line) and the V nullcline (blue points) solved numerically. The system starts at a stable fixed point and is slowly driven to cross a saddle-node and then follow a limit cycle, until it cross again a saddle-node (creating the Homoclinic bifurcation), and go back to a stable fixed point. (GIF 4822 KB) [file 10827_2022_811_MOESM2_ESM.gif]

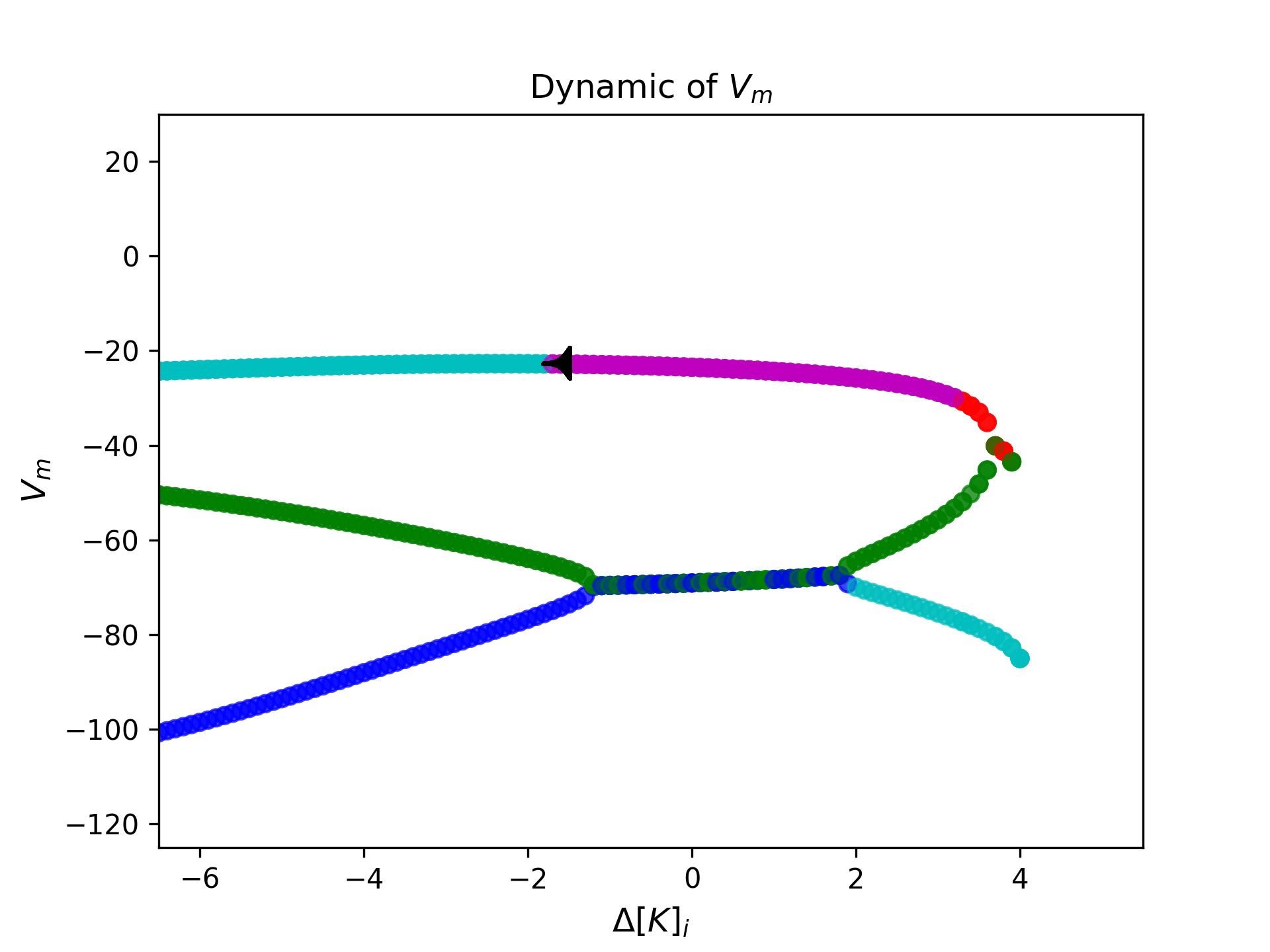

Supplement: Supplementary file 3 — Supplementary file3 S3 Animation. Dynamics of the membrane potential during SLEs. Considering the two slow variables as parameters of the fast subsystem, fixed point has been found: blue: stable node, green: saddle node, cyan: stable focus, magenta: unstable focus, red: unstable node. The system starts at a stable fixed point and is slowly driven to cross a saddle-node and then follow a limit cycle, it cross successively two Hopf bifurcations to come back to a limit cycle until it cross again a saddle-node (creating the Homoclinic bifurcation), and go back to a stable fixed point. (GIF 1305 KB) [file 10827_2022_811_MOESM3_ESM.gif]

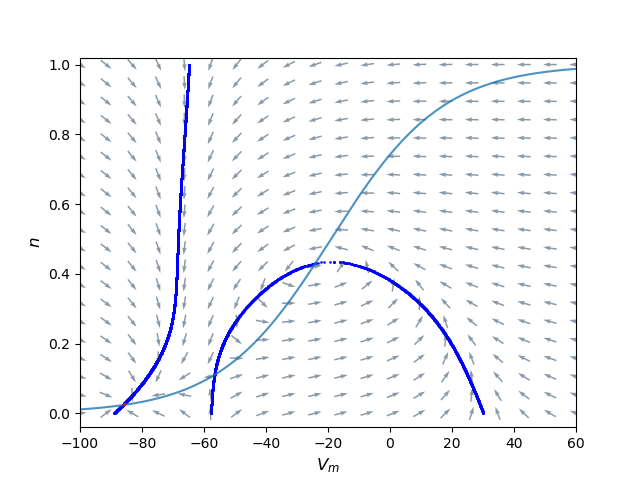

Supplement: Supplementary file 4 — Supplementary file4 S4 Animation. Dynamic during SLEs observed in the phase plane. The n nullcline (blue line) and the V nullcline (blue points) solved numerically the system start at a stable fixed point and is slowly driven to cross a saddle-node and then follow a limit cycle, it cross successively two Hopf bifurcations to come back to a limit cycle until it cross again a saddle-node (creating the Homoclinic bifurcation), and go back to a stable fixed point. (GIF 6157 KB) [file 10827_2022_811_MOESM4_ESM.gif]
